# *In Vitro* Study of Surface Modified Poly(ethylene glycol)-Impregnated Sintered Bovine Bone Scaffolds on Human Fibroblast Cells

**DOI:** 10.1038/srep09806

**Published:** 2015-05-07

**Authors:** Sumit Pramanik, Forough Ataollahi, Belinda Pingguan-Murphy, Azim Ataollahi Oshkour, Noor Azuan Abu Osman

**Affiliations:** 1 Centre for Applied Biomechanics, Department of Biomedical Engineering, Faculty of Engineering, University of Malaya, Kuala Lumpur 50603, Malaysia; 2 Department of Mechanical Engineering, Faculty of Engineering, University of Malaya, Kuala Lumpur 50603, Malaysia

## Abstract

Scaffold design from xenogeneic bone has the potential for tissue engineering (TE). However, major difficulties impede this potential, such as the wide range of properties in natural bone. In this study, sintered cortical bones from different parts of a bovine-femur impregnated with biodegradable poly(ethylene glycol) (PEG) binder by liquid phase adsorption were investigated. Flexural mechanical properties of the PEG-treated scaffolds showed that the scaffold is stiffer and stronger at a sintering condition of 1000°C compared with 900°C. *In vitro* cytotoxicity of the scaffolds evaluated by Alamar Blue assay and microscopic tests on human fibroblast cells is better at 1000°C compared with that at 900°C. Furthermore, *in vitro* biocompatibility and flexural property of scaffolds derived from different parts of a femur depend on morphology and heat-treatment condition. Therefore, the fabricated scaffolds from the distal and proximal parts at 1000°C are potential candidates for hard and soft TE applications, respectively.

The development of synthetic implants and tissue grafts mainly involves porous material integrated with biological cells or molecules for tissue engineering (TE) applications^1^. However, selection of suitable material is very critical in bone-defect repair or reconstruction applications^2^. Numerous synthetic materials, including biopolymers^3^,^4^, bioactive ceramics^5^,^6^,^7^,^8^,^9^,^10^ and high-strength composites^11^, have been tested as scaffolds. However, no synthetic material has been implanted successfully for long-term load-bearing applications because of their several disadvantages including mechanical instability, machinability, long degradation rate, and in sufficient immunogenic responses^12^,^13^. Bioceramic scaffold materials have been developed from various natural sources, including coral^14^, shell^15^ and bone^16^,^17^,^18^,^19^. The main inorganic constituent of these is calcium phosphate, which closely matches with the structure of synthetic hydroxyapatite (HA, Ca_10_(PO_4_)_6_(OH)_2_). In this way, HA nanoparticles have also been used as bioactive coating materials for surface immobilization in biosensors due to their high enzyme and protein adsorption ability, and have been employed in several biomedical applications, including prostheses and implants, using electrodeposition and various thermal spray techniques^20^,^21^,^22^. Polymers supplemented by HA have elicited intensive interest in various applications in biomedical fields^23^,^24^. Although treatment of HA with several biopolymers, such as aliphatic polyesters (e.g., polylactic acid, PLA^25^,^26^ and poly-L-lactic acid)^27^, copolymers (e.g., polylactic-co-glycolic acid)^28^, polycaprolactone^29^,^30^ and collagen^31^, has been intensively explored, these combinations have several limitations in terms of biological reaction with host tissues and mechanical strength of scaffolds. For example, lactic acid, which is a byproduct of PLA degradation, results in an adverse cellular response at the bone-implant interface by reducing the local pH, wherein human synovial fibroblasts and murine macrophages release prostaglandin^31^,^32^. On the other hand, polyol polymers e.g., poly(ethylene glycol) (PEG) have some excellent properties in TE applications^33^. The major advantage of this biodegradable polymer PEG is its nontoxicity, nonimmunogenicity, and nonantigenicity to active proteins or cells^34^,^35^. PEG can govern the cell membranes and has ability to suppress the nonspecific uptake of nanomaterials in a cell through cell membranes^36^. PEG has very little effect on surface chemistry and solubility of other molecules during attachment; however, its morphology can be controlled during attachment with other molecular surfaces^35^. Selection of MWs of PEG polymers are extremely important as its different MWs are departed from the vehicle agents in the biomineralization process during *in vivo* use in scaffolds^37^ or drug-delivery vehicles^38^. The solubility (physicochemical property) of PEG in water decreases with an increase in MW^39^. This property of PEG polymers is potentially used in drug-delivery systems^40^,^41^ and bone-tissue engineering^42^,^43^. Furthermore, bone grafting is mainly used as a potential treatment for bone-defect repair and reconstruction^8^,^44^,^45^. Scaffolds derived from bovine bone have been proven to offer potential advantages in terms of quantity and quality over synthetic or natural materials for TE scaffolds^18^,^19^,^46^. An ideal bone graft material must facilitate a substantial space for infiltrating cells to attach, proliferate, and produce a new extracellular matrix in scaffolds. Pore size and porosity of the scaffold materials are critical in bone formation^8^,^47^,^48^. Mechanical properties of the scaffolds depend on the porosity and morphological structure of the materials^6^. Matching mechanical properties and interconnected porous structure similar to the bone structure and property are highly desirable for different TE applications^6^,^7^,^12^. In our previous study^49^, we found potential differences in the morphology and chemical compositions in cortical bone along the longitudinal axis of bovine-femur. It suggests the potentiality of bovine-femoral bones as scaffolds and biomaterials. Therefore, the main objective of the present investigation is to study bovine femoral bones for developing a suitable biocompatible scaffold material having matching mechanical properties with the soft and hard tissues for TE applications. We extracted the HA or bovine apatite (BA) scaffolds from bovine cortical bones (BCBs) of different parts in a femur and impregnated with PEG by liquid phase absorption to evaluate the effects of PEG, morphology, and heat-treatment conditions on the cytotoxicity and mechanical property of TE scaffolds. Since presence of completely grown HA crystals was minimal in the bone samples sintered at 750°C (where other calcium phosphate phases had just started to convert into HA phase), as found in our previous investigations^19^,^49^, the present study focuses only on the PEG-treated BA, which were derived from the three different parts of the femoral bovine bone and sintered at 900 and 1000°C.

## Methods

### Materials

**Figure 1:**
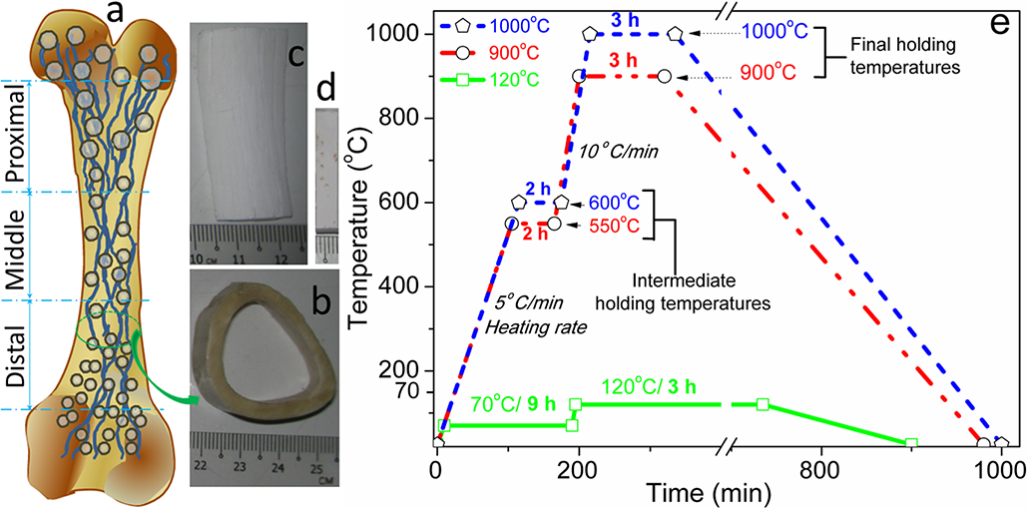
(a) A schematic image of bovine-femur with three different sections; digital images of (b) unsintered (cross-sectional view) cortical bone and (c) sintered (longitudinal view) bovine apatite before PEG impregnation and (d) PEG-impregnated sintered bovine apatite scaffold; (e) drying (green line: 120°C) and sintering conditions (red line: 900°C and blue line: 1000°C) of the samples.

**Preparation of PEG-treated bone scaffolds.** First, fresh bones from adult (aged between 2 and 3 years) bovine-femur were obtained from the local slaughterhouse. The preparation of the sintered bone scaffolds was similar to the process mentioned in our previous studies^19^,^49^. The stem part of the bovine femoral bones was sectioned along the bone length into three equal domains: proximal (near the hip joint), distal (far from the hip), and middle (between the proximal and distal), as depicted in Figure 1a. The Figures 1a–1d also depict a schematic of bovine femoral bone (Figure 1a), unsintered cross-sectional view of cortical bovine-femur (Figure 1b), sintered cortical bovine-femoral bones (Figure 1c), and PEG-treated sintered bone (Figure 1d). Extra muscles and blood from each part of the fresh bones, without any heat or chemical treatment, were entirely cleaned in boiled distilled water for 2.5 h. The cortical part of each cleaned bone, which was separated from spongy bones, was freed from extra fats by acetone (AR grade, supplied by Fischer Scientific (M)) in an ultrasonicator (SW12H, Sono Swiss) for 5 min. The BCB specimens were dried at 120°C for 12 h in an oven (Memmet, Naluri Scientific). The dried bones from different parts of the femur were sliced into proper specimens according to the test (average size for mechanical test: 3×5×22.5 mm^3^, for density, porosity, PEG volume, or biodegradation: 4×7×7 mm^3 ^and Alamar Blue, AB, assays: 3×4×5 mm^3^) and were then sintered according to the conditions shown in Figure 1e. All the BCB samples were dried at 120°C for 3 h following to intermediate holding temperature at 70°C for 9 h (see green line in Figure 1e). Then the dried samples were heat-treated by two step sintering steps at 900°C with an intermediate holding temperature of 550°C for 2h (see red line in Figure 1e) and 1000°C for 3 h with an intermediate holding temperature of 600°C for 2 h (see blue line in Figure 1e). The dried specimens were sintered separately at 900 and 1000°C, for 3 h under ambient atmosphere using box furnace (L8-1200, VISTEC Technology) to get the BA or bovine HA. Then, each BA sample was kept in the furnace until 25°C for slow cooling. The sintered specimens were reacted with boiled PEG (PEG8000, MW of 8 kDa, a specific gravity of 1.1 g/cc, supplied by Fisher Scientific (M)) by liquid phase adsorption using boiling impregnation method at an optimized condition of 130°C for 2 h in a closed chamber. The PEG was impregnated in BA by liquid phase adsorption. The whole reaction was performed in a fume hood at normal atmosphere. The specimens were then removed from the liquid PEG and immediately freed from the extra PEG, which was loosely bonded to the surface of BA by successive use of dried soft tissue paper and 5 wt% water soaked wet cotton. The liquid phase adsorbed PEG-impregnated samples were kept in a vacuum desiccator for drying at 25°C for 24 h. After drying, the sample was not sticky in normal atmospheric condition, at 25°C. After sintering at 900°C the PEG-treated BA scaffolds from distal, middle, and proximal parts are represented as PEG-D900, PEG-M900, and PEG-P900, respectively; similarly, after sintering at 1000°C the PEG-treated BA scaffolds from distal, middle, and proximal parts are represented as PEG-D1000, PEG-M1000, and PEG-P1000, respectively.

### Characterizations

**Relative density and porosity test.** Bulk densities (*ρ*, g/cc) of the scaffolds before and after PEG treatment were measured by revised Archimedes’ principle, which is especially suitable for nano- or micro-porous materials^19^,^49^. Relative density (*ρ_r_*) of the scaffolds was measured with respect to the density of pure hydroxyapatite (*ρ*_HA_ = 3.16 g/cc) following Eq. 1 and open porosity (*P*_open_, %) of the scaffolds before PEG treatment was evaluated using Eq. 2^6^,^19^,^49^. Volume percentage (vol%) of PEG in the PEG-impregnated scaffolds was determined according to Eq. 3. The PEG density was considered to be 1.1 g/cc to evaluate the vol% present in the PEG-treated scaffolds. Dissolution of PEG in water during the test was negligible amount. Since the weight change of the water in a beaker, where the PEG-treated scaffolds were tested, was less than the resolution of the weighing machine (±0.0005g), the amount of PEG or bovine HA in water was neglected. 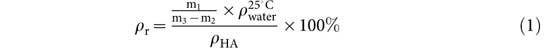


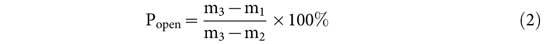


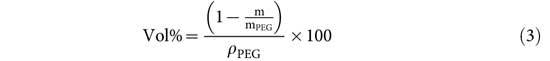
where *m*_1_ is the dry weight of the PEG-treated scaffold in air, *m*_2_ is the mass of the PEG-treated specimen in distilled water, *m*_3_ is the mass of the wet PEG-treated specimen after it is taken from the water, *m* is the dry weight of the BA scaffold before PEG treatment in air, *m*_PEG_ is the weight of the PEG-treated scaffold in air immediately after PEG treatment, and *ρ*_PEG_ is the density of PEG (i.e., 1.10 g/cc). At least five specimens of the same dimensions were examined at 25°C to evaluate the standard deviation (SD) for each sintered sample, where water density 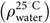 was considered to be 0.99704 g/ml.

**X-ray diffraction (XRD) technique.** Quantitative study of PEG polymer and bovine apatite (BA) ceramics phases present in the scaffold samples were determined by XRD with Cu-Kα radiation at a wavelength of λ = 1.54056Å by using a X-ray diffractometer (Empyrean, PANalytical).

**Fourier transform infrared (FTIR) spectroscopy.** Chemical functional groups present in biodegradable PEG, sintered bones (i.e., P900 and P1000), and PEG-treated sintered bone scaffolds (i.e., PEG-P900 and PEG-1000) were evaluated using attenuated total reflectance–Fourier transform infrared (ATR–FTIR) spectrometer (FTIR–ATR 400, Perkin Elmer). The spectra were collected in the range 4,000 to 450 cm^−1^ at a resolution of 4 cm^−1^.

**Flexural or three-point (3-P) bending test.** A flexural or three-point bending test is frequently employed to avoid several difficulties such as complex specimen geometry, arduous machining, and a higher chance of damaging load cell or specimen before starting the test in direct tensile and compressive tests. The main advantage of the 3-P bending test is that one surface of the testing material always undergoes compression and the reverse side experiences tension. Therefore, flexural properties obtained from a single mode testing are closely comparable to the combination of tensile and compressive properties. Thus, the 3P bending test of rectangular specimens was preferred to be conducted on the PEG-treated sintered bone scaffolds derived from three different parts of the bovine-femur in a universal mechanical tester (5848 Microtester, Instron Corporation) with a constant cross-head speed of 1 mm/min and a fixed span length of 20 mm to achieve best results. At least three specimens with same dimensions were performed at 25°C to evaluate the SD for each sintered samples.

***In vitro* degradation study.** *In vitro* degradation was tested by immersing the three sets of the PEG-treated scaffolds (PEG-D900, PEG-D1000, PEG-M900, PEG-M1000, PEG-P900, and PEG-P1000,) in the small bottle containing 15 ml of freshly prepared phosphate buffer saline (PBS, supplied by Sigma Aldrich) at pH = 7.4, 37°C and static condition for three different sets of time i.e., 1, 4 and 7 days using a shaking incubator (LSI-3016, Labtech). It is done up to 7 days since beyond that the degradation of bioceramics becomes sluggish or stagnant^50^. Dry pre-weighed three specimens from each PEG-treated scaffold were placed in separate bottles for day-1, day-4 and day-7, separately. Since no change of PBS medium was required in middle of the test, this technique eliminated the loss of any material due to the PBS medium changing. Each specimen was removed from the bottles at the predetermined times and rinsed thoroughly with distilled water followed by vacuum dried for 24 h. The degree of degradation was calculated by the percentages of weight change (Δm%) following the equation Eq. 4^51^. 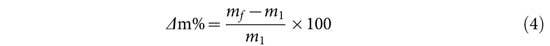 where, m_1_ is the dry weight of the PEG-treated scaffold in air before degradation and m_f_ is the dry weight after degradation test at Day-7. The surface morphology of all the PEG-treated scaffolds after degradation at Day-7 were investigated under scanning electron microscope (SEM) using dual beam focused ion beam field emission scanning electron microscope (Auriga, Carl Zeiss).

**Pore size distribution study.** Pore size distribution was performed on all the SEM images of PEG-treated scaffolds after degradation at Day-7 using Image J 1.46 software. Each SEM image after degradation at Day-7 was followed the following steps: (i) inverting the image, (ii) adjustment of brightness and contrast according to image histogram, (iii) smoothing the image, (iv) making binary, and (v) analyzing white areas.

***In vitro* cell culture study.** To check the cellular biocompatibility of the PEG-impregnated and sintered (at 900 and 1000°C) bone scaffolds from the three different femoral parts, *in vitro* cell culture test was performed on the human dermal tissue-derived fibroblast cells.

#### Cell isolation

The unused disposal dermal tissues from the gluteal region of a young female were collected after cosmetic-plastic surgery from the Medical Hospital, University of Malaya. The fibroblast cells were isolated from human skin using outgrowth method^52^,^53^. The materials (supplied by Sigma Aldrich) and technique were similar to our recent study^54^. In brief, 1 mm^2^ piece of dermal tissue was dissected mainly from the epidermal layer and rinsed with PBS repeatedly. The subculture of these dermal tissues was performed on 75 cm^2^ flasks in 10 vol% fetal bovine serum (FBS) and 1% (w/v) penicillin–streptomycin–amphotericin B supplemented high-glucose Dulbecco’s modified Eagle’s medium (DMEM) at 37°C, 5% carbon dioxide (CO_2_), 95% relative humidity (RH) conditions using a CO_2_ incubator (Forma™ Steri-Cycle™, Thermo Scientific), and at pH 7.4 to avoid non-physiologic upheavals for at least 60 days by changing culture media in every three days. The population of the harvested fibroblast cells was 2x10^6^ per flask.

#### Cell culture and Alamar Blue (AB) assay

The PEG-impregnated sintered bone samples were sterilized in absolute alcohol for 3 h at 30°C followed by autoclaving (Omega 121/134, Prestige Medical) at 2.10 kg/cm^2^ and 30°C for 20 min. Since the melting temperature of PEG was 55°C, high temperature sterilization was avoided to prevent the scaffolds from being damaged. The 200 µl human fibroblast cells of concentration 5×10^4^ cells/ml in a media of 10% bovine fetal serum and 1% penicillin–streptomycin antibiotic with high glucose DMEM were seeded to each sample in a flat bottomed 24-well plate and incubated for 4 h (according to the protocol mentioned in the instructions of supplied chemicals) to allow the cells to attach to the scaffolds using a similar optimized time protocol reported previously^55^. Positive control or simply called control (cell and medium in a 24-well plate) and negative control or simply called blank (only medium in a 24-well plate) wells were also prepared to determine the relative performance of the scaffolds. The samples and control wells were then incubated with additional 2 ml of the above DMEM solution at pH 7.4, 37°C temperature, 5% CO_2_, and 95% RH for 1, 4, and 7 days successively for cell culture in the CO_2_ incubator. The first observation was taken at day 1. The AB assay monitors the reducing environment of the living cell *in vitro*. In this quantitative colorimetric assay, the oxidation-reduction indicator AB dye is reduced to a pink-colour from a purple-blue coloured oxidized state, indicates the cytotoxicity of scaffold against the cells. The absorption was taken at wavelengths 570 nm and 600 nm to evaluate the degree of AB reduction (%) as reported elsewhere^55^,^56^,^57^. In the AB assay, after cultured on day 1, the medium was removed from each incubated sample-wells. The removed medium was free of floating cell, as observed under an optical microscope, but at the bottom of each well except negative control or blank cells were found, which were then analyzed by an optical microscopy study. Then, 2ml freshly prepared blue coloured AB solution (i.e., 10% resazurin in PBS of pH 7.4) was added to each well of the 24 well-plate to make the AB cell solution. The AB cell solution was then incubated in the same incubator at the aforesaid conditions for 4 h to obtain a stable-cellular purple solution. The incubated 100 µl AB cell solution was then transferred in to a 96-well microplate and placed in a microplate reader (FLUOstar Optima, BMG Labtech) within 5 min to measure the light absorption at two different wavelengths of 570 and 600 nm. For continual assessment of cell proliferation, the same day-1 samples were washed by PBS two times and then made to undergo the AB assay until day-4 and day-7, respectively. At least three samples with same dimensions were tested for each test. It was observed that after day-7, the fibroblast cells had grown very fast and formed a layered structure on top of the other cell-layers on the scaffolds. This can cause cell-death due to the overwhelming cell population^4^. The AB assay was used to check the cytotoxicity through direct contact with cells in terms of cell viability property of the materials on fibroblast cells. This technique is an indirect method for assessing cell growth and proliferation quantified by a microplate reader because mitochondria oxidize the resazurin solution (i.e., purple blue at oxidized state) of AB, resulting in a pink end product at reduced state. The amount of colour absorption by the living cells is attributed to the amount of metabolic activity or cell proliferation. Results were expressed as the mean for each triplicate culture. Thus, the absorbance by cells corresponds to a percentage of reduction of AB (%AB reduction) is directly proportional to a larger number of proliferating cells. The percentage of reduction of AB (%AB reduction) was calculated by following Eq. 5^56^,^57^. 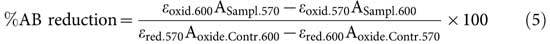 where molar extinction coefficients of AB in the oxidized and reduced controls are 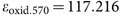 and 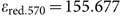, respectively at wavelength of 570 nm; 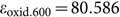 and 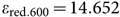 are molar extinction coefficients of AB in the oxidized and reduced controls, respectively at wavelength of 600 nm^58^; and 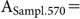 absorbance of the scaffold samples at 570 nm, 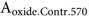 = absorbance of the oxidized negative controls at 570 nm, 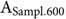 = absorbance of the scaffold samples at 600 nm, and 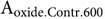 = absorbance of the oxidized negative controls at 600 nm. The population of cells or cell concentration (i.e., (1 + AB reduction) × initial cell) was evaluated from the %AB reduction with respect to the initial cell concentration used in this study, assuming that all the incubated cells had taken part in reducing the AB to produce a homogeneous AB-cell solution.

**Optical microscopy.** Cell morphology, migration, proliferation, and distributions of the live cells, which had detached from the scaffolds and distributed on a 24-well plate, were studied under an optical microscope (Eclipse TS 100, Nikon) after days 1, 4, and 7 of the AB assay. Since the cells were seeded only on to the scaffolds for incubation, these cells migrated and proliferated only from the scaffolds to the well.

**Scanning electron microscopy.** PEG-impregnated scaffolds after AB assayed only for day 7 was investigated to check the cell attachment and cell migrations into the three-dimensional (3D) scaffolds from its surface with different morphologies of the differently treated materials. In the present study, no other chemical was used to fix the cells on the scaffold. After Day-7 of AB assay, the cell attached scaffold samples were collected and directly dried in vacuum at 25°C for 48 h. The dried samples were then broken by vertical chiselling technique to observe the cell migrations into the scaffolds under SEM. Since the scanning electron microscopy was performed on the broken cross-section of the scaffolds, all the cells were assumed to have migrated from the surface through the interconnected pores. Cell attachment was determined by the visible adherence of the cells to scaffold’s inner surface from the SEM images.

**Statistical analysis.** A two-way ANOVA was employed to find the impact of different treatments on scaffolds along with cell culture time on mechanical, physical, and biological properties at 95% confidence. The critical level of null hypothesis was tested by f-test distribution by considering a probability value of p < 0.05 to evaluate the significant difference in density, impregnated PEG volume in BA, change in weight in *in vitro* degradation, AB absorption, and AB reduction between the scaffolds with different treatments and culture time.

## Results

**Relative density and porosity test.** Relative density and open porosity of the only sintered bovine bones (before PEG treatment) and the PEG-impregnated scaffolds (after PEG treatment to the sintered bovine bone) and amount of PEG present in PEG-impregnated scaffolds are illustrated in Table 1. The relative density (*ρ*_r_), open porosity (*P*_open_, %), and PEG-volume percentage (V_PEG_, %) were evaluated using Eqs. 1, 2, and 3, respectively. Results in Table 1 showed that the relative density of all the PEG-impregnated scaffolds was significantly (p = 0.005) higher than that of untreated BA scaffolds. At 900°C sintering condition, the relative density was lowest for all scaffolds derived from the three parts of the same bovine-femur compared with the 1000°C sintering condition (see Table 1). By contrast, the open porosity of the untreated scaffolds closely matched with the corresponding adsorbed volume (%) of impregnated PEG present in the PEG-treated scaffolds and the volume of PEG was slightly higher than the open pore volume of the untreated BA scaffolds. The open-pore volume in the BA scaffolds sintered at 900°C before PEG impregnation, was comparable with the impregnated PEG volume present in the PEG-treated scaffolds sintered at 900°C, and was higher than those of the 1000°C sintering condition for all three regions of the same bovine-femur.

**Figure 2:**
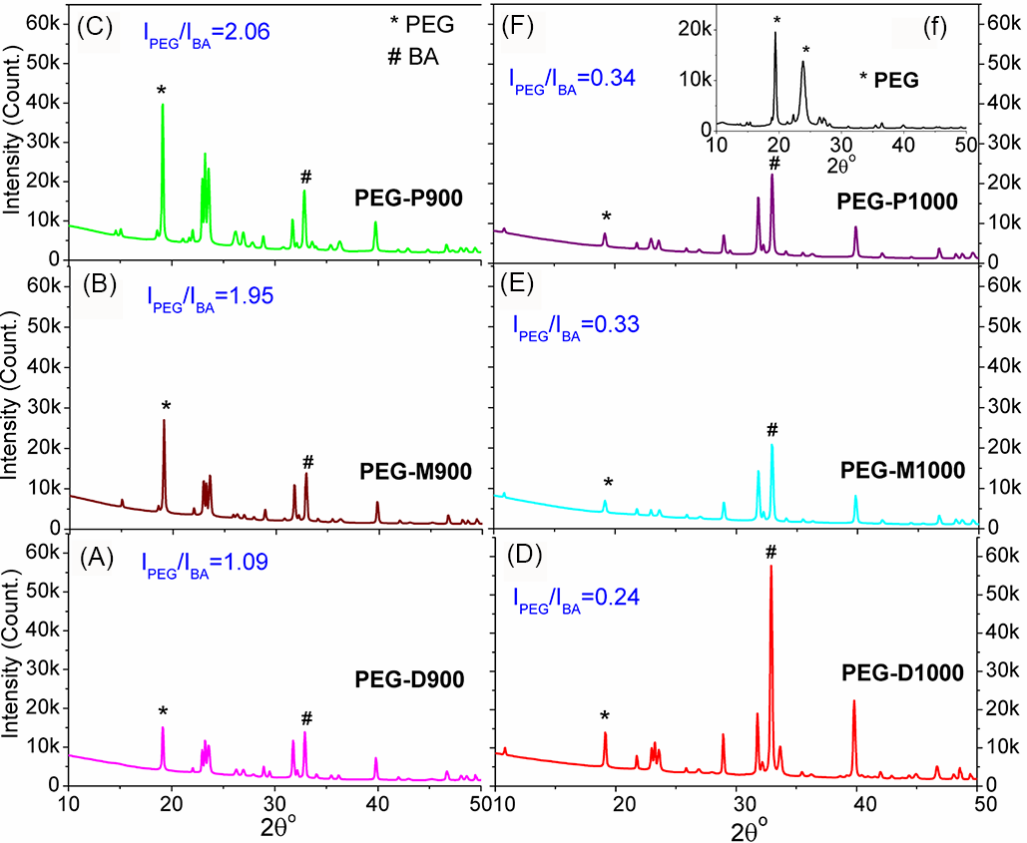
XRD patterns of the PEG-impregnated bovine apatite (BA) scaffolds: (A) PEG-D900, (B) PEG-M9000, (C) PEG-P900, (D) PEG-D1000, (E) PEG-M1000, and (F) PEG-P1000; inset (f) XRD pattern of pristine PEG. Intensity ratio (I_PEG_/I_BA_) of PEG (I_PEG_) to BA (I_BA_) correspond to their maximum intensities (semicrystalline peak of PEG ‘*’ and crystalline peak of BA‘#’) indicates the quantitative presence of PEG in BA.

**XRD technique.** XRD patterns of the PEG impregnated BA such as PEG-D900, PEG-M9000, PEG-P900, PEG-D1000, PEG-M1000 and PEG-P1000 are depicted in Figures 2A–2F, respectively. The XRD pattern of pristine semicrystalline PEG is also depicted as inset in Figure 2f. The peaks around 2θ = 19° and 23° were two major identification peaks of PEG. These two peaks were also found in all the PEG-BA scaffolds in Figures 2A–2F. The main identification peaks (211), (112) and (310) of BA were found at around 2θ = 31.7°, 32.8° and 39.9°, respectively and it is resembled with the standard hydroxyapatite (JCPDS 09-0432)^49^. Ratio of highest intensity (I_PEG_) peaks of PEG at 2θ = 19°, as marked by star ‘*’, and BA (I_BA_) at 2θ = 32.8°, as marked by hash ‘#’, are depicted in Figure 2. The maximum intensity of the PEG and BA and their ratios are illustrated in Table 2. The intensity ratio, I_PEG_/I_BA_, was indicative of relative amount of impregnated PEG in the PEG-treated scaffolds. The I_PEG_/I_BA_ ratio was highest for the PEG-treated scaffold, which was made from proximal part of bovine femur and lowest for distal part specimens at both sintering conditions. Sintering temperature also affected on the I_PEG_/I_BA_ ratio as scaffolds prepared at 900°C has higher values compared to 1000°C.

**Figure 3:**
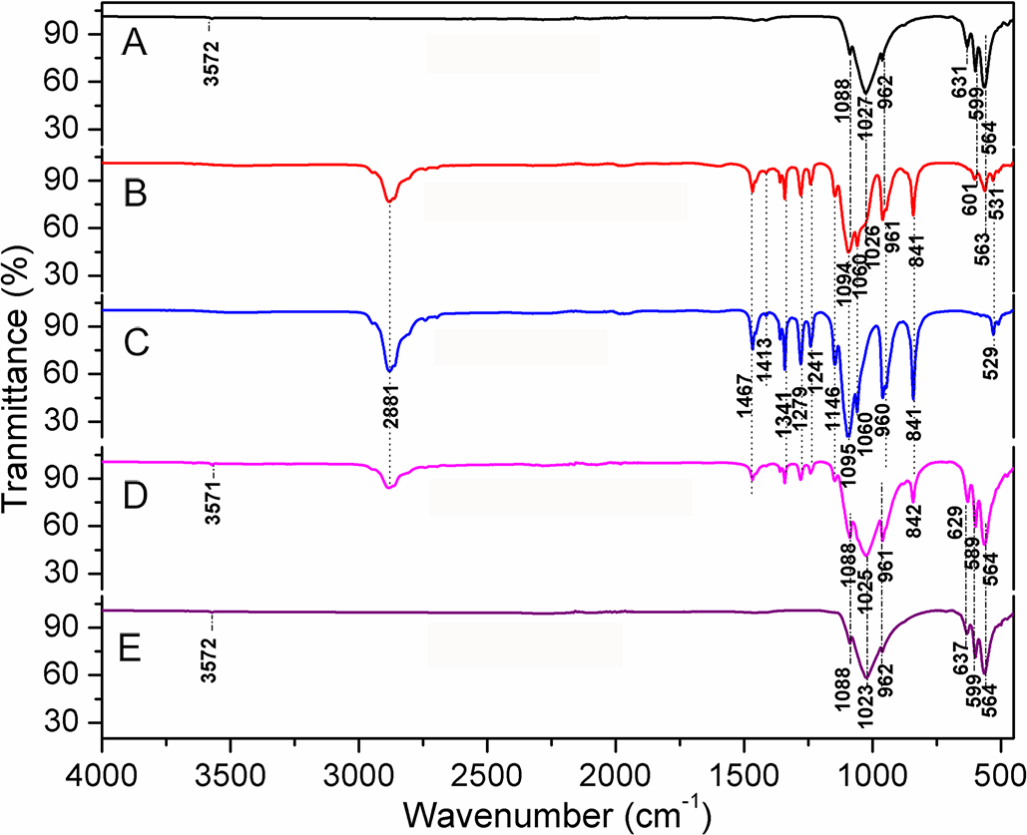
FTIR spectra of (A) BA scaffolds sintered at 900°C, (B) PEG-impregnated BA scaffolds sintered at 900°C (PEG-P900), (C) pristine PEG, (D) BA scaffolds sintered at 1000°C, and (E) PEG-impregnated BA scaffolds sintered at 1000°C (PEG-P1000). Note that most of the peaks of PEG and the untreated BA are present in PEG-P900 and PEG-P1000, indicating the strong adsorption of PEG in the scaffolds.

**FTIR spectroscopy.** Presence of PEG in the sintered BA was confirmed by FTIR spectroscopy. FTIR spectra of BA sintered at 900°C, PEG-P900, pristine PEG, BA sintered at 1000°C, and PEG-P1000 are depicted in Figures 3A–3E, respectively. In Figure 3, the PEG-impregnated scaffolds showed that they contained all the functional groups those were present in the sintered BA (i.e., P-O, C = O, molecular O-H) and PEG (i.e., C-C, C-O-C, alcoholic O-H, and C-H). In the present study, the corresponding FTIR peaks of PEG, illustrated in Table 3, were found in Figure 3C (blue line). Functional groups present in the sintered bone materials (i.e., P900 and P1000) are similar to the pure HA, which was already explored in our previous report^49^. It has been found that most of the peaks of PEG and the untreated BA are present in PEG-P900 and PEG-P1000. It indicates the adsorption of PEG in the scaffolds is substantial.

**Figure 4:**
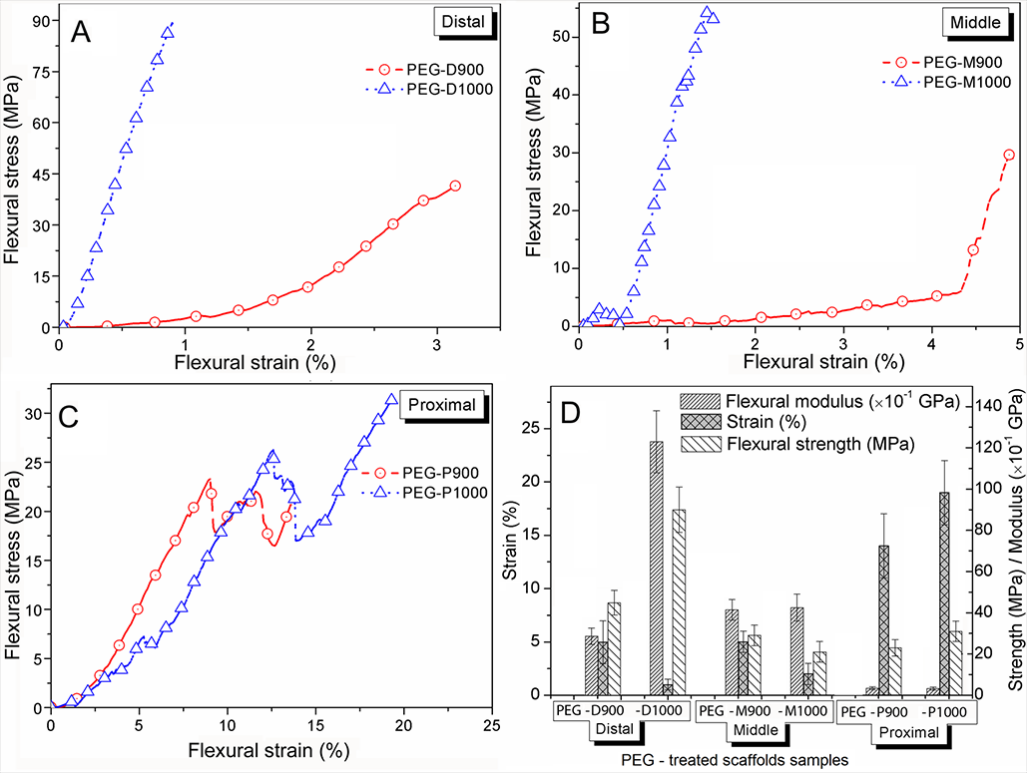
(A – C) Stress–strain behaviors and (D) comparisons in strength, modulus, and strain under 3P-bending mode for the PEG-impregnated sintered bone scaffolds derived from the three different parts (A – distal, B – middle, C – proximal) of a bovine-femur. Flexural strength and modulus is higher for all the scaffolds sintered 1000°C but flexural strain is higher only for the scaffolds proximal part.

**Flexural or 3-P bending test.** Figure 4 shows stress–strain behaviors and comparisons in strength, modulus, and flexural strain at failure under 3P-bending mode for all liquid phase adsorbed PEG-impregnated samples. The flexural strength of the PEG-treated scaffolds sintered at 1000°C was higher compared with 900°C sintering condition. The modulus of the PEG-treated scaffolds sintered at 1000°C were higher and flexural strain was lower in the sample from the distal and middle parts compared with the samples derived from the proximal part.

**Figure 5:**
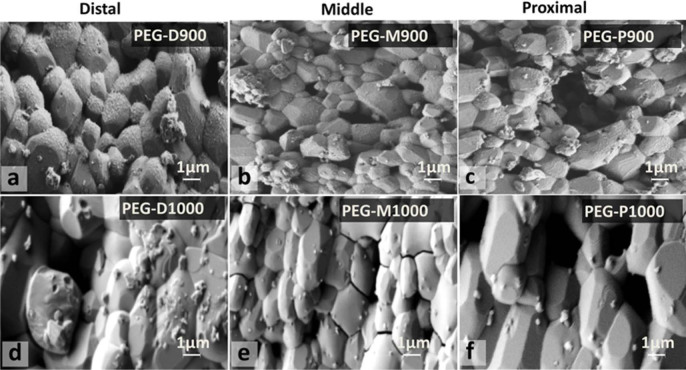
Surface morphology of PEG-treated BA scaffolds (a – PEG-D900, b – PEG-M900, c – PEG-P900, d – PEG-D1000, e – PEG-M1000, and f – PEG-P1000) after degradation test for day-7 at 37°C in PBS. Presence of PEG in a–c compared to d–f, but size of deposited particles is large in d–f compared to a–c. PEG impregnated sintered bovine scaffolds on Days 1, 4, and 7.

***In vitro* degradation study.** Degradation result of PEG-treated scaffolds illustrated in Table 4 showed that all the scaffolds degraded up to day-4, and then the weight increased at day-7. The SEM images were depicted in Figure 5 for the PEG-treated scaffolds after day-7 to investigate the surface morphology.

**Figure 6:**
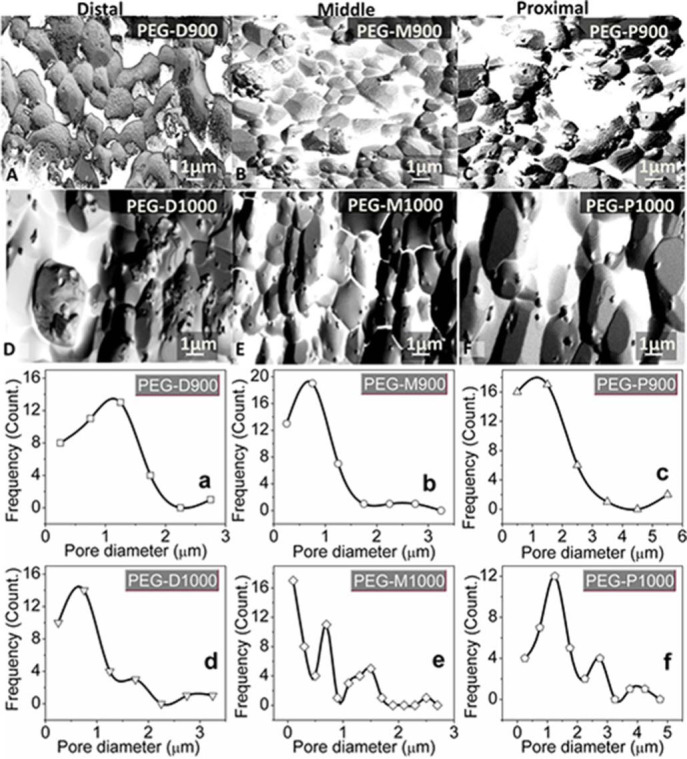
Inverted SEM images of PEG treated BA scaffolds (A – PEG-D900, B – PEG-M900, C – PEG-P900, D – PEG-D1000, E – PEG-M1000, and F– PEG-P1000) after degradation test for day-7 at 37°C in PBS. Pore size distribution of these scaffolds using image J software are for (a) PEG-D900, (b) PEG-M900, (c) PEG-P900, (d) PEG-D1000, (e) PEG-M1000, and (f) PEG-P1000, respectively. Note: single mode pores are present in the PEG-treated scaffolds sintered at 900°C and multimode pores at 1000^°^C. Scaffolds from proximal part have wide range of pore size compared to distal or middle part of the bovine femur.

**Pore size distribution study.** Pore size distribution of was evaluated on the PEG-impregnated scaffolds after biodegradation at day-7 after image processing in Image J software. The white colour in the Figures 6A–6F represents the pore areas. The size of each area was converted to equivalent diameter in the software. The corresponding size distribution was plotted in the Figures 6a–6f, respectively.

**Figure 7:**
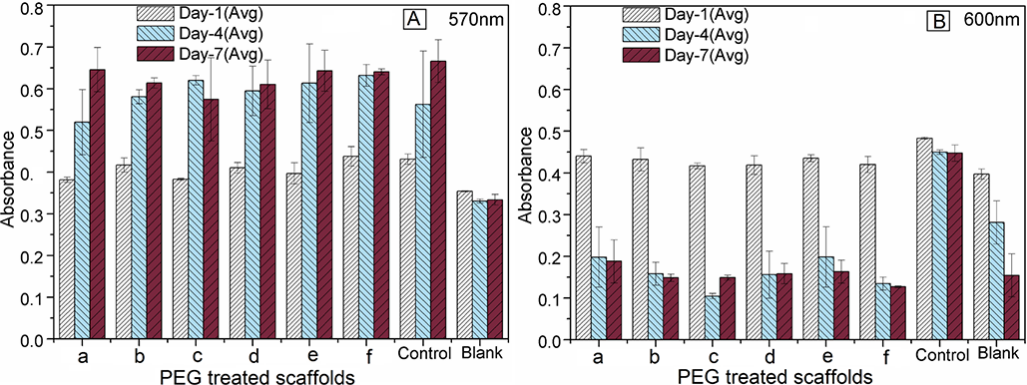
Alamar Blue cell absorbance properties of PEG-impregnated BA scaffolds (a – PEG-D900, b – PEG-D1000, c – PEG-M900, d – PEG-M1000, e – PEG-P900, and f – PEG-P1000), control and blank at wavelengths of (A) 570 nm and (B) 600 nm. Note that the absorbance values increases significantly (p<0.001 for both wavelengths) with culture time from day 1 to day 7 at lower wavelength.

**Figure 8:**
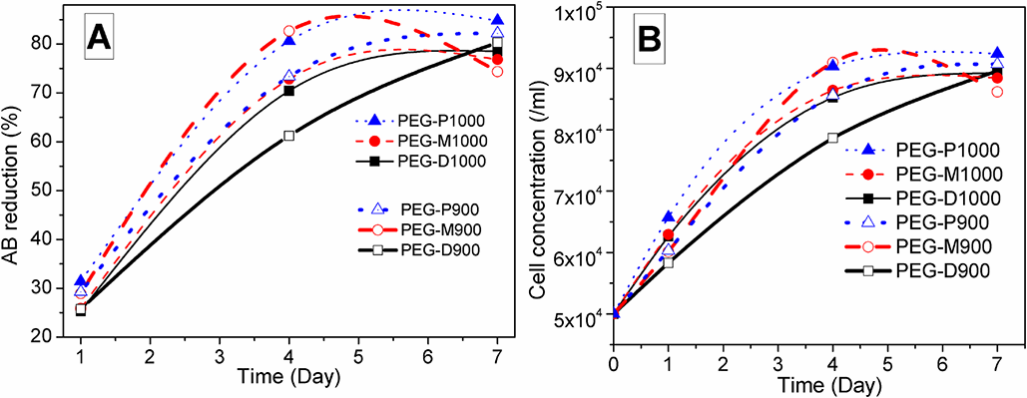
A) Alamar Blue reduction (%) behavior of PEG-impregnated BA scaffolds (for distal – square, middle – round, proximal – triangular) sintered at 900 and 1000°C in AB assay using 570 nm and 600 nm wavelengths. AB Reduction increases with time and it is highest for PEG-P1000 scaffold at day 7. B) Cell population or concentration varies with the PEG-impregnated BA scaffolds (symbols used for distal – square, middle – round, and proximal – triangular) sintered at 900 and 1000°C.

***In vitro* AB assay.** AB assay measured fibroblast cell proliferation of the scaffolds using light absorption at two wavelengths: 570 nm (Figure 7A) and 600 nm (Figure 7B). The absorbance deduced for all the scaffolds and positive controls was measured by the respective absorbance of negative controls or blanks, which showed the absorption only for the DMEM medium. The absorptions of the blank wells are also shown in Figure 7. AB reduction (%) indicates the amount of fibroblast cell proliferation by detecting the level of oxidation and it is distinctly depicted in Figure 8. AB assay was mainly used to determine the AB reduction (%) of the light absorption at 570 nm and 600 nm wavelengths for oxidized and reduced AB, respectively following Eq. 5. The biocharacteristics of PEG-treated bovine cortical bone scaffolds sintered at 900 and 1000°C are depicted in Figure 8A. The population of cell was evaluated corresponding to the %AB reduction in depicted in Figure 8B.

**Figure 9:**
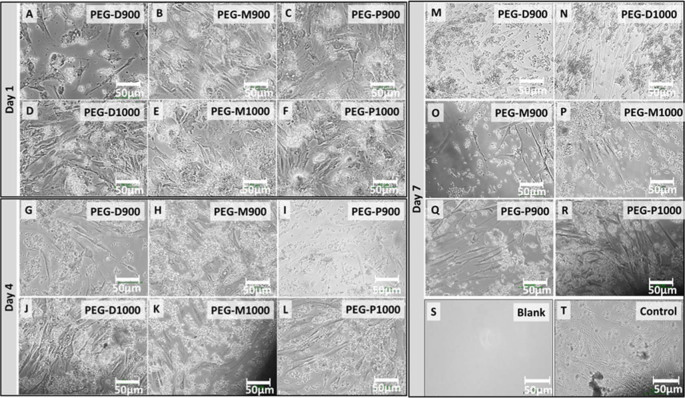
Optical micrographs of the live fibroblast cells at wells adjacent to the PEG-impregnated BA scaffolds sintered at 900 and 1000°C derived from the three different femoral sections at day-1 (A – PEG-D900, B – PEG-M900, C – PEG-P900, D – PEG-D1000, E – PEG-M1000, and F – PEG-D1000), day-4 (G – PEG-D900, H – PEG-M900, I – PEG-P900, J – PEG-D1000, K – PEG-M1000, and L – PEG-D1000), and day-7 (M – PEG-D900, N – PEG-D1000, O – PEG-M900, P – PEG-M1000, Q – PEG-P900, and R – PEG-P1000) just before the AB assay. Images of (S) negative control or blank (only medium in well, without cell and scaffold) and (T) positive control (only medium and cells in well, without scaffold) wells after day 7 are presented for comparison. All the scale bars are 50 µm. Amount of live cell concentration is higher in the wells for the PEG-treated scaffolds sintered at 1000°C compared to 900°C.

**Optical microscopy.** Optical micrographs of the live human fibroblast cells at the bottom surface of wells adjacent to the PEG-impregnated BCB scaffolds sintered at 900 and 1000°C are depicted in Figures 9A–9S. Optical images of blank and positive control wells up to day-7 are also presented in Figure 9S and 9T for comparison with the scaffolds containing wells.

**Figure 10:**
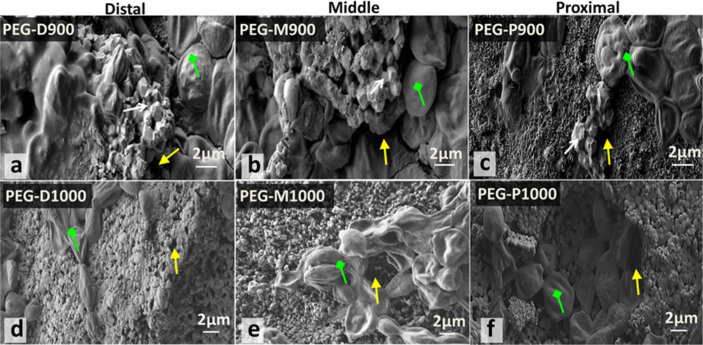
SEM morphology of the broken surface of PEG-impregnated BA scaffolds sintered at 900 and 1000°C derived from three different femoral sections with cells and morphology of the fibroblast cells that adhered to the PEG-treated scaffolds (a – PEG-D900, b – PEG-M900, c – PEG-P900, d – PEG-D1000, e – PEG-M1000, and f – PEG-P1000) after day 7 of AB assay. Attachment of the cell or cell colony (green colured arrow) is found inside the scaffolds and it is very high for PEG-P1000. Sharp edged particles, pores, and cells are indicated by white, yellow, and green coloured arrows, respectively.

**Scanning electron microscopy.** SEM images in Figure 10 showed the PEG impregnated scaffolds adhered with cells after AB assay for 7 days to illustrate the morphology and migrations of cells in the PEG-impregnated BCB scaffolds sintered at 900 and 1000°C derived from the three bovine femoral sections (distal, middle, and proximal).

## Discussion

**Relative density and porosity test.** The relative density (see Table 1) of all the PEG-impregnated scaffolds is significantly (p = 0.005) higher compared to the untreated scaffolds. This is because of a noticeable amount of PEG, which was impregnated in the porous scaffold during liquid phase impregnation process. Since the impregnated volume of PEG is slightly higher than the open pore volume of the untreated scaffolds, it strongly indicates that PEG can occupy all the open pores and additionally, PEG can create more interconnected channels through which it can reach to some close pores of sintered bone scaffolds owing to the driving force generated by impregnation process. Higher open-pore volume and impregnated PEG volume in the scaffolds sintered at 900°C compared to the scaffolds sintered at 1000°C further affected on the mechanical properties of scaffolds. Furthermore, this result occurred, because a higher amount of polymers was burnt, which were present at the proximal domain of the original as-received femoral bone. Since cortical bovine bone is a natural composite of inorganic ceramics and organic polymers, low melting temperature polymers were gradually removed with sintering temperatures. According to our previous study^49^, the proximal part of the femoral bovine bone contains the highest amount of polymers, which mainly consist of collagen fibrils.

**XRD technique.** XRD result depicted in Figure 2 indicates the quantitative amount of PEG in the PEG-impregnated BA scaffolds. Higher value of the intensity ratio (I_PEG_/I_BA_) indicates the higher amount of impregnated PEG presents in the PEG-treated BA scaffolds. The I_PEG_/I_BA_ ratio in Figure 2 implies that the PEG-treated scaffolds sintered at 900°C have higher PEG content in comparison with the PEG-treated scaffold sintered at 1000°C. Thus, sintering temperature has effect on the PEG content. It has also been noticed that PEG content is increased for the scaffolds prepared from distal (D900 or D1000) to proximal (P900 or P1000).

**FTIR spectroscopy.** In Figure 3, the PEG-treated bone scaffold (PEG-1000) had shown a shift of PO_4_^3-^ vibration peak at 629 cm^−1^ from 637 cm^−1^ along with a loss of peak at 1413 cm^−1^ due to symmetric CH_2_ stretching of PEG. A superimposition of asymmetric PO_4_^3−^ stretching peak at 1027–1026 cm^−1^ (for sintered bone P900) and 1025–1023 cm^−1^ (for sintered bone P1000) of bovine-HA with the alcoholic C-OH peaks of PEG at 1095–1060 cm^−1^ are a clear indication of chemical interaction between bovine-HA and PEG polymer. All the functional groups present in PEG, BA, and PEG-treated BA scaffolds as revealed in FTIR spectra are also illustrated in Table 3. Molecular OH of bovine-HA also formed a weak bond with PEG chains; and as a result, the peak at 3572 cm^−1^ of BA sintered at 900°C was eliminated in the PEG-P900 sample. This is because at high temperature, the oxygen of alcoholic OH of the liquid PEG becomes more polar which helps these ionic PEG polymer chains to bind with hydroxyl O-H of the bovine HA by an ionic bond (Ca_5_(PO_4_)_3_–O**^δ^**^–^**H**^δ+^**…^δ^**^–^**O**H^δ+^–R–OH). Thus, PEG acts as binder with the bovine-HA.

**Flexural or 3-P bending test.** In Figure 4, the flexural strength was found to be lower for the PEG-treated scaffolds derived from every part of the bovine-femur sintered at 900°C (i.e., PEG-D900, PEG-M900 and PEG-P900). This result is ascribed to the high porosity formed in BCB at the sintering condition of 900°C as found in the previous study^49^. This result is also supported by another study, which showed that mechanical strength deteriorates with increasing of porosity^6^. The higher modulus value was shown by the PEG-treated scaffolds sintered at 1000°C derived from the distal and middle parts compared to the proximal part. In contrast to the other HA composites reported in elsewhere^59^,^60^,^61^, the modulus values of the PEG-treated scaffolds sintered at 1000°C in the present study are substantially higher. Modulus of the few scaffolds (PEG-D900, PEG-M900, and PEG-M1000) was matched with the human trabecular bone^62^, whereas PEG-D1000 showed near to human cortical bone^63^. On the other hand, modulus of the PEG-P900, and PEG-P1000 matched with the human soft tissues^64^,^65^. Our present finding indicates that the mechanical strength of the PEG-treated scaffolds from all the three parts at 1000°C sintering condition is higher owing to the dense microstructure (see Figures 5d & 5e) having larger relative density (see Table 1) compared to the other sintering condition at 900°C. On the other hand, flexural failure strain of the PEG-treated scaffolds of 900°C was higher for the distal and middle parts compared to the 1000°C sintering conditions owing to the more amount of PEG contained. However, as observed, the scaffolds derived from the proximal region showed lower strain at 900°C (14% for PEG-P900) compared with those at the sintering condition at 1000°C (19% for PEG-P1000). This result is attributed to the excess pores, which present as a wide range of interconnected pores, in the PEG-treated scaffolds sintered at 1000°C (PEG-P1000) compared with the scaffolds derived from the proximal domain at 900°C (PEG-900). This finding is observed owing to the influence of PEG in the scaffold of the proximal domain at 900°C, where highest amount (21 vol%) of impregnated PEG was observed (see Table 1). The impregnated PEG also increased the flexural modulus compared with the proximal domain at 1000°C. In addition, as seen in Figures 4C and 4D, at 900°C, the PEG-treated scaffolds derived from proximal part showed 64% higher strain (14% for PEG-P900) than that of the other two parts (5% for PEG-M900 or PEG-D900). This is occurred owing to the excess pores, which enabled better binding and adsorption of PEG inside the scaffolds. It is indicated by the impregnated PEG volume, which was 32% higher in PEG-P900 than that of PEG-P1000. Thus, the influence of PEG in the sintered BCB scaffolds greatly effects on their mechanical properties. In contrast to the mechanical strength of the dried bone from the three different parts of a bovine-femur in the previous study^49^, the PEG-impregnated scaffolds from the proximal part show lowest flexural strength and were shown highest from the distal section. Consequence of more unidirectional collagen fibril polymers has different effect, was found in the dried BCBs compared to the present strengthening mechanism. In the present study, the bovine-HA particles of PEG-impregnated scaffolds from the proximal part were bonded with excess amount of semicrystalline PEG particle by liquid phase adsorption^66^.

***In vitro* degradation study.** The *in vitro* degradation data of the biopolymer PEG impregnated BA scaffolds revealed that the weight change with the tested time (1, 4, and 7 days) is significant (p = 0.02). The effect of temperature or impregnated PEG polymer volume has more significant (p = 0.002) effect on the weight change of the scaffolds. It clearly indicates that impregnated PEG volume, which was changed with sintering conditions of the scaffolds as well as impregnation process, has great effect on the *in vitro* biodegradation in PBS. In Figure 5, the SEM images revealed the typical surface morphology of the PEG-impregnated scaffolds after biodegradation at day-7. All the grain surfaces of the scaffolds sintered at 900°C (PEG-D900, PEG-M900, and PEG-P900) are homogeneously coated with impregnated PEG in addition to some deposited crystals. On the other hand, the scaffolds sintered at 1000°C (PEG-D1000, PEG-M1000, and PEG-P1000) are more clear with less amount of PEG on the surface but large size deposited crystals. It also indicates the impregnated PEG was more homogeneously distributed in the scaffolds sintered at 900°C compared the scaffolds sintered at 1000°C. The deposited crystals on both scaffolds are from the PBS solution which might be influenced by dissolved calcium ion (Ca^2+^) of BA, because calcium phosphates were also found to be dissolved in different medium by other studies^9^,^67^. Therefore, the mechanical properties of the scaffolds deteriorate after biodegradation, as found by different researchers^50^.

**Pore size distribution study.** Figures 6A to 6F depict the porous morphology of inverted image of the PEG-treated scaffolds after degradation at day-7. The pore sizes distribution of the scaffolds evaluated from the images Figures 6A to 6F is depicted in Figures 6a to 6f, respectively. The scaffolds sintered at 900°C contained mainly single modal pores having maximum pores of 1.1, 0.7, and 1.2 µm for PEG-D900, PEG-M900, and PEG-P900, respectively and while the scaffolds sintered at 1000°C contained multimodal pores. Pore size range in the scaffold derived from distal and middle parts was 100 nm to 3.2 µm, whereas this range was noticeably higher for proximal part samples (200 nm to 5.5 µm). Pore size distribution results of the present study also resembled with the other bioceramics^10^.

***In vitro* cell culture study.** In Figure 7, the absorbance is higher at lower (excitation) wavelength (570 nm) compared with higher (emission) wavelength (600 nm). Initially, at day-1, the difference in absorbance was insignificant (p = 1.4 for wavelength 570 nm and p = 1.7 for wavelength 600 nm) for all the scaffolds with respect to their positive controls. It indicates that the cytotoxity variation within the scaffolds were not significant. At higher wavelength, the absorbance decreased with cell-culture time, whereas at lower wavelength, the absorbance values were substantially high and also significantly increased with culture time. The latter result indicates the significant (p<0.001 for both wavelengths) growth of fibroblast cells on the scaffold samples with culture time from day 1 to day 7. Moreover, excellent biocompatibility of all PEG-treated bone scaffolds is suggested.

The amount of AB reduction (%) in colour absorption by live fibroblast cells corresponds to cell proliferation is depicted in Figure 8A. Thus, the significant (p = 0.01) increased AB reduction% with culture time (from day-1 to day-7) is indication of a larger number of proliferating cells. The trend in cell population was also similar to the %AB reduction as depicted in Figure 8B. This result also strongly supports the AB absorbance results. The AB reduction and cell concentration characteristics of the AB assay on the scaffolds suggest that rate of cell growth properties became sluggish after day-7 as the maximum rate of AB reduction (%) or cell growth was found at day-4 study. Thus, based on the AB assay results, the percentage of AB reduction% was largest at day-7, which indicates greater cell proliferation because of stimulation with PEG-impregnated scaffolds sintered at 1000°C. An exception is observed for distal PEG-treated scaffolds at day-7 due to dense microstructure of the PEG-D1000. Moreover, a higher biocompatibility was observed for the PEG-treated scaffolds those were sintered at 1000°C compared with those sintered at 900°C. Although it was not statistically significant (p = 0.4) at a particular time line, the difference indicates that only the porosity and pore size are not factors for cell growth in a biomaterial. Further, it was confirmed by morphology study in SEM, which showed that particle shape is also another important factor for cytotoxicity or cell viability. Most importantly, the scaffolds sintered at 1000°C from the proximal region showed maximum reduction compared with the others owing to the more interconnected porosities provided by the PEG-treatment. Therefore, this study indicates that the cell viability also depends on interconnected pores, which were developed by PEG-treatment, beside over all porosity.

**Optical microscopy.** In Figure 9 depicts the live cells which had not attached to scaffolds and migrated from scaffolds to the well-plate surface. As observed, cell concentration was higher in the PEG-treated BA scaffolds those were sintered at 1000°C compared with those sintered at 900°C for all three femoral sections. One of the main reasons for this result is the sharp edge of the grains scaffolds at 900°C sintered condition which inhibit the growth and lifespan of cells. Furthermore, cell concentration was higher for the scaffolds derived from the proximal section at both sintering conditions compared to the other femoral parts. This result shows all the scaffolds were nontoxic and is a supportive upshot of the AB assay.

**Scanning electron microscopy.** The morphology of the scaffold-substrate in the present study closely resembles the scaffolds derived from a femoral BA sintered at 900 and 1000°C before PEG-impregnation, as reported previously^49^. The cells growing in all PEG-treated scaffolds tended to form colonies/aggregates (see green coloured arrow in Figure 10). Thus, the difference in cell morphology from live cells, as shown in optical microscopy, was related to the effect of PEG in AB solution with time, and it is in agreement with other studies^68^,^69^. Cell migrations were better in scaffolds sintered at 1000°C (e.g., PEG-P1000) compared with those sintered at 900°C despite higher porosity present in the scaffolds at 900°C (e.g., PEG-P900), as depicted in Table 1. This was confirmed by the SEM Figure 10, where more amount of cells were present on the cross-sectional surface of the PEG-treated scaffolds sintered at 1000°C (Figures 10d–10f) compared with 900°C (Figures 10a–10c) and occurred due to the scaffolds sintered at 900°C contained more sharp-edged grains or particles, as indicated by white coloured arrows in the SEM micrographs in Figures 10a–10c. By contrast, at 1000°C, the grains or particles become much smoother, exhibit dense structures but having a wide range of more interconnected pores (see yellow coloured arrows) in Figures 10d–10f. In addition, the multimodal pores of these scaffolds were also seen by pore size distribution result (see Figure 6d–6f). Therefore, besides overall porosity, the cell migration or proliferation also depends on the interconnected pores, and shape and surface properties of the particles or grains present in the scaffolds. An evident of this argument was explored in another study wherein cell viability was found to depend on the particle shape^70^.

## Conclusions

In this study, bovine cortical bones from different parts of a femur were heat-treated and integrated with PEG by liquid phase adsorption to design a potential 3D TE scaffold. The biodegradable PEG acted as a binder with BA to keep strength of the scaffold structure. Given the biodegradable property of PEG, it can also improve cell proliferation and mechanical properties. The quantitative analysis of all PEG impregnated scaffolds was analyzed by XRD and qualitative study on the scaffolds from proximal part was employed by FTIR. The present investigation on the compositional studies by XRD and FTIR also strongly supports the previous studies^46^,^49^. Flexural strain at failure of the scaffolds is higher for the proximal femoral part because of the presence of more interconnected pores, which enable PEG to better reach inside the scaffolds. The interconnected porosity is confirmed by the impregnated PEG-volume, which was higher than the open pore volume of the scaffolds (see Table 1). The excess impregnated PEG volume also indicated that the new interconnected channels were created during PEG adsorption by liquid phase adsorption and hence influenced the flexural strain, strength and modulus by facilitating polymer matrix within the BA ceramics. It has been found that the PEG-treated scaffolds sintered at 1000°C with multimodal pore distribution have better cell response than those of higher porosity scaffolds sintered at 900°C. Thus, the *in vitro* cell culture study, including AB assay and microscopy, of the scaffolds on human fibroblast cells strongly suggests that the cytotoxicity or cell growth property of a biomaterial not only depends on porosity or pore size but interconnection between the pores as well as shape and surface properties of the particles. The scaffolds derived from the proximal domain are more flexible, which facilitates better cellular response. On the other hand, scaffolds derived from distal part have highest flexural strength and modulus. Moreover, PEG-treated scaffolds from a suitable part of bovine-femur at both 1000°C and 900°C sintering conditions would be potential candidates for hard or soft TE applications depending on the desired range in mechanical properties as well as cytotoxicity^62^,^63^,^64^,^65^. Therefore, the fabricated scaffolds derived from the distal part at 1000°C and proximal part at 1000°C would be potential candidates for hard and soft TE applications, respectively.

## Figures and Tables

**Table 1:** Bulk density and porosity of the scaffolds before the PEG impregnation and density, volume of PEG, mechanical properties of PEG-impregnated sintered bone.

PEG impregnated sintered bovine bone scaffold	Relative density of untreated sintered BA, before PEG treatment±SD	Relative density of PEG soaked sintered bone±SD	Open porosity in sintered bone±SD	Volume of impregnated PEG in sintered BA±SD (SD: standard deviation)
	%	%	%	%
PEG- D900	42.72±0.95	58.86±0.22	10.28±1.45	13.91±0.04
PEG- D1000	51.27±0.63	60.57±0.32	4.90±0.01	7.85±0.59
PEG- M900	42.09±1.27	56.99±0.28	15.08±2.3	17.88±0.09
PEG-M1000	50.95±2.85	58.61±0.32	9.23±1.78	9.57±1.04
PEG-P900	41.14±1.89	55.28±0.44	18.68±3.82	21.66±0.45
PEG-P1000	50.63±1.89	57.63±0.13	10.38±1.62	14.69±0.57

**Table 2:** XRD data analysis for the dried PEG impregnated sintered bovine scaffolds.

PEG impregnated specimens	XRD peak intensity counts for		Intensity ratio for samples	
	PEG polymer phase (I_PEG_)	Bovine apatite ceramic phase (I_BA_)	Sintered at 900°C	Sintered at 1000°C
	at 2θ = 19.2°	at 2θ = 32.8°	[I_PEG_/I_BA_]_900°C_	[I_PEG_/I_BA_]_1000°C_
PEG-D900	15088	13895	1.09	
PEG-D1000	14028	57555		0.24
PEG-M900	27021	13848	1.95	
PEG-M1000	6945	20843		0.33
PEG-P900	36641	17757	2.06	
PEG-P1000	7507	22330		0.34

**Table 3:** FTIR peak positions of the all functional groups present in the PEG and PEG-treated bovine apatite.

Name of bond	Peak position (cm^−1^) in the materials		
	PEG	Untreated proximal BA sintered at 900 and 1000°C	PEG-P900 and PEG-P1000
Molecular OH	Absent	3572	3572
Symmetric CH_2_ stretching	2881		2881
C-H (i.e., alkane) bending and scissoring C-H (i.e., alkane)	1467, 1413, and 1341		1467, 1413, and 1341
C-O and C-C stretching in the crystalline phase	1279		1279
Asymmetric C-O-C stretching	1241		1241
Symmetric C-O-C (i.e., ether) stretching	1146		1146
C-O (i.e., alcoholic) stretching	1095		1095
CO-C axial deformation	1060	1060	1060
Asymmetric PO_4_^3-^ stretching		1023–1027	1023–1027
PO_4_^3-^ stretch symmetric		962	962
= C-H bending	960	960	960
C-CH aliphatic deformation vibration	841		Absent
PO_4_^3-^ vibration		637	Absent
PO_4_^3-^ vibration		Absent	629
PO_4_^3-^ vibration		564, 599	564, 599
C-C vibration	529		Absent

**Table 4:** Biodegradation test data of the PEG impregnated sintered bovine scaffolds at 37°C in PBS on Days 1, 4, and 7.

PEG impregnated scaffold	Weight change ± SD (%)		
	Day-1	Day-4	Day-7
PEG- D900	−9.13±0.02	−12.66±0.07	+6.65±0.12
PEG- D1000	−5.49±0.05	−8.49±0.13	+5.63±0.08
PEG- M900	−11.09±0.09	−15.84±0.08	+6.92±0.13
PEG-M1000	−7.24±0.08	−9.13±0.06	+6.38±0.06
PEG-P900	−12.37±0.07	−19.03±0.13	+8.90±0.08
PEG-P1000	−10.13±0.06	−11.13±0.14	+6.71±0.11
